# A whole-genome worldwide molecular epidemiology approach for contagious caprine pleuropneumonia

**DOI:** 10.1016/j.heliyon.2020.e05146

**Published:** 2020-10-08

**Authors:** Etienne Loire, Abdoulkarim Issa Ibrahim, Lucía Manso-Silván, Louis Lignereux, François Thiaucourt

**Affiliations:** a CIRAD-ASTRE, Montpellier, France; b OIE/FAO World Reference Laboratory for CCPP, France; c INRA, UMR1309 ASTRE, Montpellier, France; d Tillaberi University, BP 175, Tillaberi, Niger; e LABOCEL, BP 485, Niamey, Niger; f Research Unit for Epidemiology and Risk Analysis Applied to Veterinary Sciences (UREAR-ULiège), Centre of Fundamental and Applied Research for Animals and Health (FARAH), Faculty of Veterinary Medicine, University of Liège, Liège, Belgium; g School of Animal and Veterinary Sciences, University of Adelaide, Roseworthy, 5371, South Australia, Australia

**Keywords:** Bioinformatics, Genetics, Microbiology, Evolutionary biology, Veterinary medicine, Contagious caprine pleuropneumonia, *Mycoplasma capricolum* subsp. *capripneumoniae*, Whole genome phylogeny

## Abstract

Contagious caprine pleuropneumonia is an infectious and contagious disease affecting goats and wildlife ruminants, mostly in Africa and Asia. It is caused by a mycoplasma, *Mycoplasma capricolum* susbp. *capripneumoniae*, which is very fastidious. This may be the reason why there are few reports of its isolation and characterization. This study describes the development of a whole genome typing strategy based on sequencing reads assemblies on a reference genome (Abomsa, GenBank accession LM995445) and extraction of informative single nucleotide polymorphism. FASTA sequences inferred from the variant calling files were used to establish a comprehensive phylogenetic tree based on 2880 SNPs. This tree included a total of 34 strains originating from all the regions where CCPP has been detected, as well as strains isolated from wildlife. A recent isolate from West-Niger was positioned closely to another 1995 East-Niger isolate, an indication that CCPP may be extending westward in Africa. Six 2013 Tanzanian isolates had identical sequences in spite of diverse geographical origins. This could be explained by the clonal expansion of a virulent strain at that time in East Africa. Although all strains isolated from wildlife in the Middle East were in the same phylogenetic group, this may not sign an adaptation to new hosts. The most probable explanation for wildlife contamination remains the contact with goats. This strategy will easily accommodate new data in the near future and should become a gold-standard high-resolution typing procedure for the surveillance of contagious caprine pleuropneumonia.

## 1 Introduction

During the early days of bacterial genome sequencing, one mycoplasma was chosen as an example, the agent of contagious bovine pleuropneumonia (CBPP) *Mycoplasma mycoides* subsp. *mycoides* (Mmm). The reasons for this choice: because mycoplasmas are the simplest self-replicating organisms possessing very small genomes (Westberg et al., 2004). Unfortunately, these scientists picked the wrong species as Mmm genome is riddled with repetitive elements such as insertion sequences (Frey et al., 1995; Vilei et al., 1999). This made their task daunting and lengthy, owing to the sequencing tools available at the time. The situation has dramatically changed today now that it is viable to sequence dozens of bacterial genomes simultaneously. It is even easier with bacterial genomes that do not possess mobile genetic elements and are not prone to horizontal gene transfer (Sirand-Pugnet et al., 2007). This is the case with the agent of contagious caprine pleuropneumonia (CCPP), *Mycoplasma capricolum* subsp. *capripneumoniae* (Mccp), as revealed by the few complete genomes published so far (Chu et al., 2011; Dupuy and Thiaucourt, 2014) (Yu et al., 2013; Falquet et al., 2014) (Li et al., 2020). Whole genome sequencing (WGS) therefore offers a unique opportunity to expand our knowledge on the epidemiology of CCPP.

Contagious caprine pleuropneumonia is a disease notifiable to the World Organization for Animal Health (OIE) that affects goats and many wild ruminant species. Its known distribution spans from North-Central Africa to China (Manso-Silvan and Thiaucourt, 2019). As such, it is a threat for millions of goats raised in low-income countries and it dramatically affects the livelihood of many poor households. The only CCPP vaccine validated so far is based on a bacterin (Rurangirwa et al., 1987). It is difficult and expensive to produce, when respecting OIE guidelines. As a result, good quality vaccines are almost never used in regions where CCPP is highly prevalent, for technical as well as financial reasons (Thiaucourt et al., 2018) and goat owners resort frequently to antibiotic treatments in case of an outbreak.

A clinical and anatomo-pathological diagnosis of CCPP is theoretically easy, as typical lesions are observed in acute outbreaks (Nicholas and Churchward, 2011). The presence of unilateral pleuropneumonia lesions had readily been observed when CCPP was described in the literature for the first time in Algeria (Thomas, 1873). But the occurrence of pneumonia in goats is usually attributed to other causes, such as Peste des Petits Ruminants (PPR) or « Pasteurellosis » and laboratory confirmation of CCPP outbreaks is very seldom achieved although specific diagnostic tests exist (Woubit et al., 2004; Peyraud et al., 2014; Liljander et al., 2015; Settypalli et al., 2016). The isolation of Mccp is not frequent and very rarely reported, first because field samples are often not of adequate quality and second because Mccp is one of the most fastidious mycoplasmas (MacOwan and Minette, 1976) (Nicholas and Churchward, 2011). Isolation requires quality assured media and experienced technicians. Consequently, very few CCPP outbreaks are confirmed nationally and notified to the OIE, and the true distribution and prevalence of CCPP remain largely unknown.

The objective of this study was to establish a whole-genome-based characterization of Mccp strains that may supersede previously developed strategies for molecular epidemiology studies, from single-locus (Lorenzon et al., 2002) to multi-locus (Manso-Silvan et al., 2011) and then large-scale, based on next generation sequencing (NGS) data (Dupuy et al., 2015). A genomic analysis pipeline was applied to a representative number of Mccp strains including isolates spanning all regions where CCPP is present. Efforts were also targeting the finishing of a number of Mccp whole genome sequences. Once established, the usefulness of this procedure was evaluated with a number of recent Mccp field strains originating from west Niger, Tanzania or strains isolated from wildlife in the United Arab Emirates.

## 2 Materials and methods

### 2.1 Mccp genome sequencing

A set of 34 Mccp strains was chosen from the CIRAD-ASTRE bank (Table 1). It included strains originating from the various regions where CCPP is known to be present and, notably, five strains isolated from wildlife (wild goat from Qatar (*Capra aegagrus*) (Arif et al., 2007), and Arabian sand gazelle (*Gazella marica*) (Lignereux et al., 2018), Arabian oryx (*Oryx leucoryx*) (Chaber et al., 2014) and scimitar-horned oryx (*Oryx dammah*) from the United Arab Emirates. It also included six strains isolated in various locations in Tanzania in 2013, a year of high CCPP prevalence (E. Noah, personal communication). Strain Gabes was passaged in vitro up to 102 times as to evaluate the effect of in vitro passages on genetic drift. Passages 22 and 102 were included in the study. Ten ml of cultures were centrifuged at 12000g for 25min, the pellet was resuspended in 200μl of PBS, and DNA was extracted using the Qiagen blood and tissue kit and sent to Macrogen (Seoul, Republic of Korea) for DNA sequencing. Genomic libraries (Illumina TruSeq DNA PCR Free) were constructed and then analysed by Illumina HiSeq 2500 (rapid run mode 2X250bp sequencing lane). Genome assemblies were performed with Seqman NGen (V14) using the genome of Mccp strain Abomsa (GenBank accession LM995445) as a reference. The assemblies were first performed with a single genome representative of each of the six clusters formerly identified by a large-scale genomic analysis/a sample of 57 genes (Dupuy et al., 2015). Inconsistencies were resolved by analysing individual flanking reads, which were used to evaluate if the inconsistency was due to a deletion or an insertion. After manual correction of the genome sequences, the assemblies were reiterated until no inconsistency remained. Each of these initial genomes was then used as reference for the assembly of the genomes of strains belonging to the same cluster. Completed genomes were sent to the NCBI for automatic prokaryotic annotation (Haft et al., 2018) and publication.

**Table 1:** List of *Mycoplasma capricolum* subsp. *capripneumoniae* strains or genomes used in that study. A total of 34 strains and 5 Mccp genomes were included in this study. They are grouped according to their phylogenetic position which is in agreement with the grouping established previously (Dupuy et al., 2015). Their geographical localization spans the known CCPP distribution and their isolation dates varies from 1958 to 2019. Strain 87001 has been described as isolated in 1958 (Li et al., 2020), about 20 years earlier than the F38 strain isolated in Kenya (MacOwan and Minette, 1976), but the number of in vitro passages is unknown for that strain.

Group	CIRAD_ID	strain	collected_by or published_by	collection_date	geo_loc_name	host	lat_lon	GenBank acc.
A	97095	97095	Salam Tariku	1988	Ethiopia: Tigray	*Capra hircus*	13.87 N 39.00 E	
	98112	M79/93	Dr G Bölske	1995	Uganda: Karamoja	*Capra hircus*	3.32 N 34.06 E	
	14015	14015	Dr E Noah	2013	Tanzania: Bagamoyo	*Capra hircus*	-6.43 N 38.88 E	
	14019	14019	Dr E Noah	2013	Tanzania:Simanjiro	*Capra hircus*	-4.28 N 37.11 E	
	14020	14020	Dr E Noah	2013	Tanzania: Kiteto	*Capra hircus*	-5.85 N 36.89 E	
	14022	14022	Dr E Noah	2013	Tanzania: Kongwa	*Capra hircus*	-6.20 N 36.41 E	
	14025	14025	Dr E Noah	2013	Tanzania: Korogwe	*Capra hircus*	-5.17 N 38.44 E	
	14026	14026	Dr E Noah	2013	Tanzania: Manyoni	*Capra hircus*	-5.77 N 34.79 E	
	ILRI181	ILRI181	Dr L Falquet	2012	Kenya: Laikipia	*Capra hircus*	0.40 N 36.78 E	LN515399
	04012	04012	Dr Arif	2004	Qatar	***Capra aegagrus***	25.34 N 51.17 E	CP040917∗
	16034	16034	Dr L. Lignereux	2016	United Arab Emirates	***Oryx dammah***	24.12 N 55.83 E	
	13092	13092	Dr L. Lignereux	2013	United Arab Emirates	***Gazella marica***	24.12 N 55.83 E	
	14001	14001	Dr AL Chaber	2014	United Arab Emirates	***Oryx leucoryx***	24.12 N 55.83 E	
	14003	14003	Dr AL Chaber	2014	United Arab Emirates	***Oryx leucoryx***	24.12 N 55.83 E	
B	94156	438LP	Dr P Hendrikx	1994	Chad: N'Djamena	*Capra hircus*	12.17 N 15.00 E	CP041708∗
	05021	05021	Dr M Niang, Swasou Abbas	2004	Sudan: Nyala	*Capra hircus*	12.05 N 24.88 E	CP041700∗
	8789	8789	Dr S Buron	1987	Chad: Karal	*Capra hircus*	10.42 N 16.94 E	
	19146	19146	Dr A Issa-Ibrahim	2019	Niger: Tahoua	*Capra hircus*	14.90 N 5.22 E	
	95043	95043	Dr Y Maikano, Dr Thiaucourt	1995	Niger: Goure	*Capra hircus*	14.03 N 10.25 E	CP041705∗
C	91106	C550/1	Dr U Wernery	1991	United Arab Emirates	*Capra hircus*	25.08 N 55.27 E	CP041703∗
	11022	44F04	Dr U Ozdemir	2011	Turkey: Thrace	*Capra hircus*	41.26 N 27.07 E	
	12002	12002	Dr Amirbekov	2011	Tajikistan	*Capra hircus*	37.89 N 69.17 E	CP041702∗
D	M1601	M1601	Dr Chu	2007	China: Gansu	*Capra hircus*	38.28 N 101.46 E	CP017125
E	9081	487P	Dr H Said Al Sumry	1990	Oman	*Capra hircus*	23.42 N 58.35 E	
	07033	033C1	Dr B Cetinkaya	2007	Turkey: Elazig	*Capra hircus*	38.67 N 39.35 E	CP041712∗
	Gabes	Gabes	Dr P Perreau	1982	Tunisia: Gabes	*Capra hircus*	33.89 N 10.07 E	
	Gabes 22p∗∗∗							
	Gabes 102p∗∗∗							
	LKD	LKD	Dr P Perreau	1982	Tunisia: Kebili Douz	*Capra hircus*	33.47 N 9.05 E	
	8891	8991	Dr G.E. Jones	1986	Oman	*Capra hircus*	22.59 N 57.80 E	CP041701∗
F	F38	F38	Dr K J MacOwan	1976	Kenya∗∗	*Capra hircus*	-0.91 N 36.81 E	LN515398
	89110	AMRC-C758	Dr M S Harbi	1983	Sudan	*Capra hircus*	15.22 N 32.31 E	CP041711∗
	97017	Yatta/B	Dr H Wesonga	1997	Kenya: Yatta	*Capra hircus*	1.18 S 37.53 E	CP041707∗
	Abomsa	Abomsa	Dr F Thiaucourt	1982	Ethiopia: Godjam	*Capra hircus*	10.52 N 37.81 E	LM995445
	92138	CLP1	Dr J Fikre	1992	Ethiopia: Bishoftu	*Capra hircus*	8.77 N 38.99 E	
	91039	2/90	Dr F Thiaucourt	1991	Ethiopia: Awash	*Capra hircus*	9.01 N 40.09 E	CP041710∗
	94029	C5	Dr G J King	1994	Oman	*Capra hircus*	23.42 N 58.35 E	CP041709∗
	97097	Erer	Salam Tariku	1997	Ethiopia: Erer	*Capra hircus*	9.56 N 41.37 E	CP041706∗
G	87001		Dr Li	1958	China: Shandong	*Capra hircus*	35.96N 116.90 E	CP006959
H	zly1309F		Dr Yu	2012	China: Nagqu	***Pantholops hodgsonii***	31.69 N 91.50 E	CP019061

∗ This study.

∗∗ Pecise location unknown.

∗∗∗ Gabes strain was passaged in vitro up to 102 times.

### 2.2 Core genome analysis

Snippy pipeline (https://github.com/tseemann/snippy) was used to analyse complete genome sequences present in the NCBI database and raw sequence data generated from the CIRAD-ASTRE biobank. For FASTA sequences previously published ((Dupuy and Thiaucourt, 2014) (Falquet et al., 2014) (Yu et al., 2013) (Li et al., 2020)), pseudo reads were generated to keep a homogeneous methodology. For each sample, reads were mapped on the genome sequence of strain Abomsa with “bwa-mem” (Li and Durbin, 2009), keeping only reads uniquely mapped with a quality above 60 (Phred score). Duplicates were filtered with samtools (Li et al., 2009) and soft clipped positions removed with samclip (https://github.com/tseemann/samclip). Variants were called using freebayes (Garrison and Marth, 2012), filtering variants with less than 10X coverage and a calling quality of 100. Additional filtering steps were taken to remove variants called from read extremities. FASTA sequences were inferred from the variant calling files and core genome alignments were produced with snp-sites (Page et al., 2016). Maximum-likelihood phylogenetic trees were inferred from core genome alignments using FastTree (Price et al., 2010) with a GTR + gamma model. Shimodaira-Hasegawa tests (SHT) were used to assess the local support values of the nodes. Tree visualization was done using iTOL (Letunic and Bork, 2019) or Figtree (http://tree.bio.ed.ac.uk/software/figtree/).

### 2.3 CCPP field outbreak in Niger

In October 2019, a CCPP suspicion was observed near Tahoua, 350km North-East from Niamey, in a herd of 16 sahelian goats (*Capra hircus*) previously vaccinated against PPR. Six animals suffered from acute respiratory distress with signs of nasal discharge, cough and painful breathing. A 3-month-old kid subsequently died. It had extensive lung lesions and a piece of hepatized lung was sent to LABOCEL, Niamey, for analysis. Minced pieces of hepatized lung were seeded in a modified Hayflick liquid medium (Thiaucourt et al., 1992b), which comprised an autoclaved PPLO broth base and a filtered supplement. The classical supplement composition (30% horse serum, 5% fresh yeast extract, 2 g/l glucose, 4 g/l pyruvic acid and 0.1 g/l ampicillin) was enriched with 10 mg/l rifampicin (Rifadine, Sanofi) and 2.5 mg/l amphotericin B (Sigma A9528) to further prevent bacterial and fungal contaminants. When growth was observed, after 6 days of incubation at 37 °C, 10 ml of culture were centrifuged at 12,000g for 20 min and the pellet resuspended in 200μl of phosphate buffered saline solution (PBS). DNA was extracted from this suspension using the DNeasy Blood and Tissue kit (Qiagen). The presence of Mccp was established by 16 rDNA PCR (Botes et al., 2005) and sequencing of the amplified product (IAEA Vienna Austria). Electrophoregrams were analysed (DNASTAR SeqMan Pro) and the final sequence was analysed by BLASTn (https://blast.ncbi.nlm.nih.gov/) on 12/11/2019. The DNA, identified as *M. capricolum* species, was sent to CIRAD-ASTRE laboratory, OIE/FAO reference laboratory for CCPP.

## 3 Results

**Figure 1:**
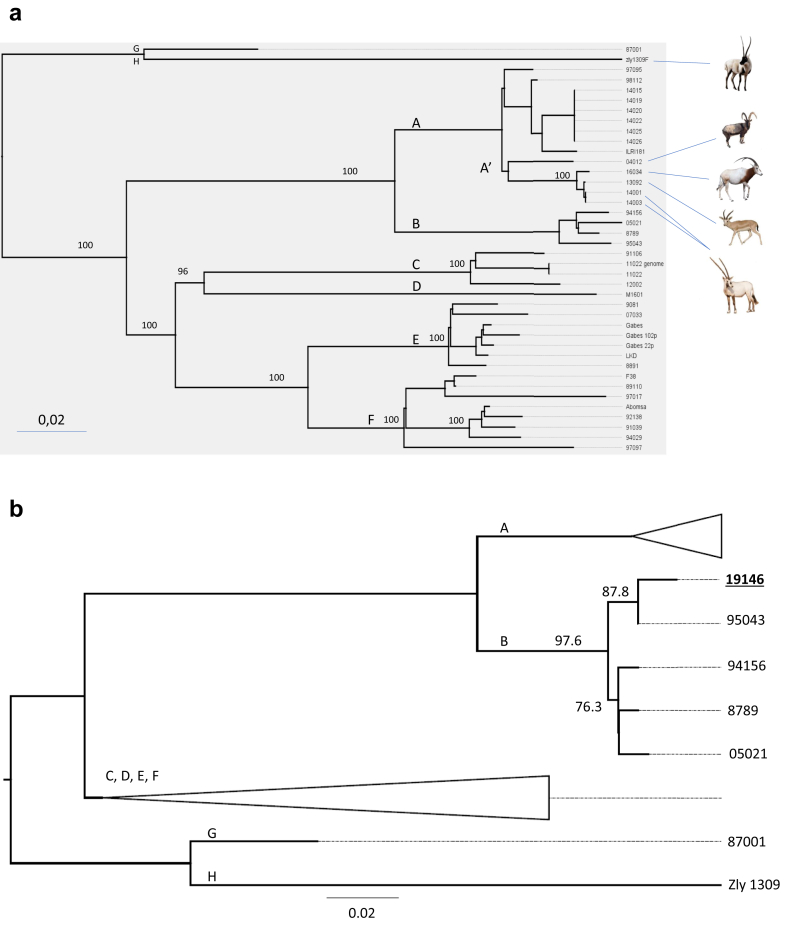
Phylogenetic trees inferred from *Mycoplasma capricolum* subsp. *capripneumoniae* core genome SNPs analysis. a. This tree was constructed by analyzing a set of Mccp genomes retrieved from the NCBI website or Illumina reads from 34 Mccp strains. It is based on a total of 2880 SNPs with all nodes leading to the various phylogenetic groups being supported by 100 Shimodaira-Hasegawa test values. The two samples “11022”, obtained from Illumina reads, and “11022 genome”, obtained by creating pseudo-reads from a finished genome, positioned at the same tip of a branch. The effect of the *in vitro* passage of strain Gabes can be visualized by the positions of Gabes-22p and Gabes-102p on various branches whose length is proportional to the number of passages. Wild animal silhouettes correspond to: *Pantholops hodgsonii, Capra aegagrus, Oryx dammah, Gazella marica and Oryx leucoryx*. (from top to bottom). Phylogenteic groups A,B,C,D,E,F were inferred from Dupuy et al. (Dupuy et al., 2015), groups A’, G and H were created during this study. b. As the number of reads available for sample 19146 was much lower, this second tree was based only on 396 SNPs. Nevertheless, it allowed the reconstruction of a robust phylogenetic tree. The two samples from Niger, 95043 and 19146 are positioned on a branch supported by 87.9 Shimodaira-Hasegawa test value. The longer branch for 19146 materializes the genome evolution of two strains isolated at 24 years interval.

**Figure 2:**
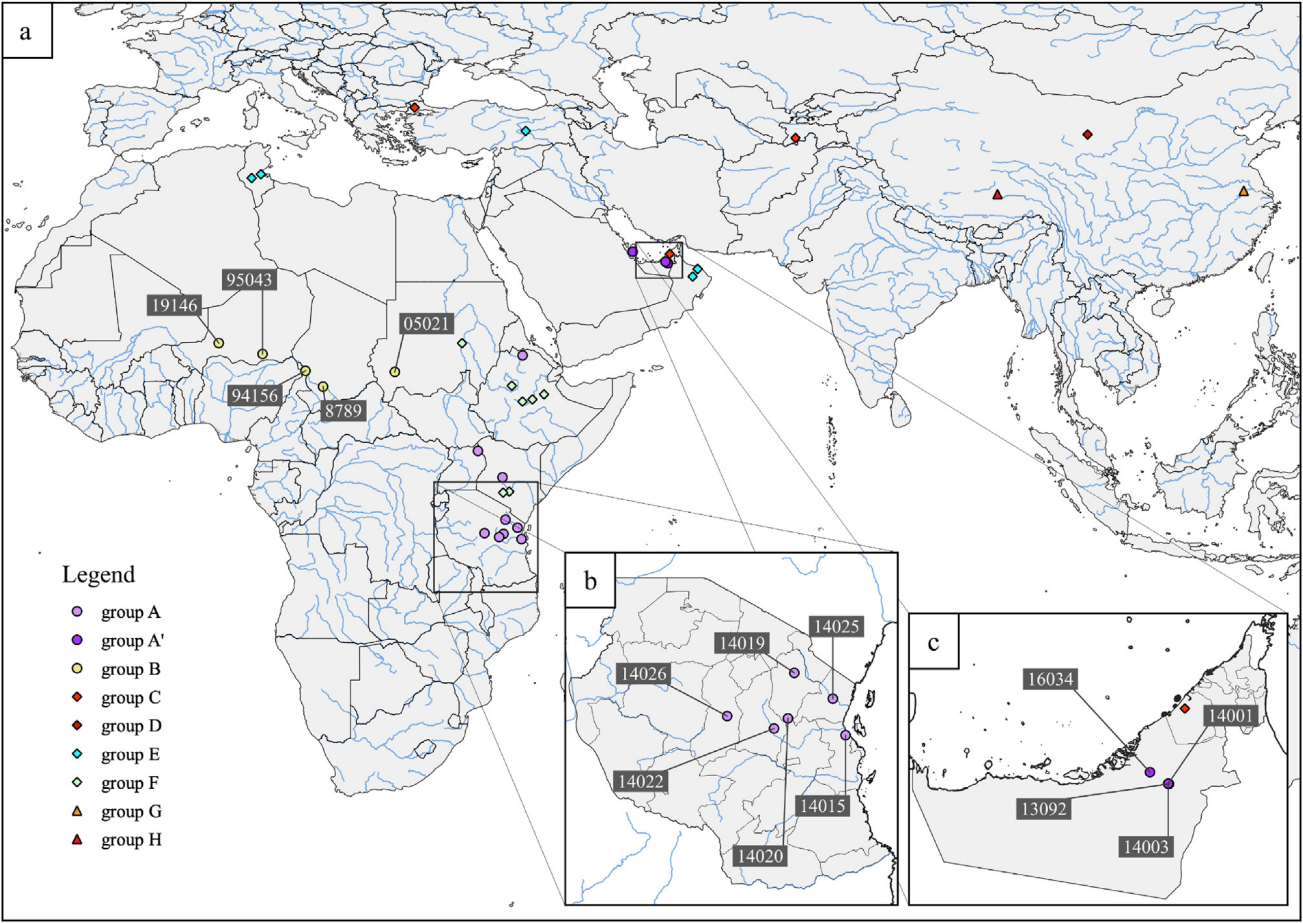
Geographic origins of the strains analyzed in this study. a. Strains are represented by symbols corresponding to their phylogenetic grouping. They are positioned at their sampling locations except for strains 87001 and F38 whose exact origins are unknown. Strain 19146 is the most western strain in Sub-Saharan Africa. b. All six Tanzanian Mccp strains isolated in 2013 had exactly the same genome sequence in spite of various geographical origins. c. Conversely, Mccp strains (16034) isolated from wildlife 31km apart from others were clearly distinguishable. This is in favor of separate contamination sources.

The core genome pipeline analysis of all 34 samples, based on the Abomsa reference genome, yielded 2880 SNPs, which were the basis for the construction of a robust phylogenetic tree (Figure 1a) as well as a geographical representation of analysed strains (Figure 2a).

All nodes leading to the phylogenetic groups were supported by 100 SHT values. The six 2013 isolates from Tanzania shared the same sequence and positioned within cluster A, with the closest relative being ILRI181 isolated in Kenya in 2012. The five strains isolated from wildlife in the Arabic Peninsula grouped together and formed a sub-cluster, A’. Strain 16034, isolated from an *Oryx dammah*, had a significantly different sequence from the other wildlife isolates from the United Arab Emirates although they were obtained in two locations 31km apart. A single SNP was identified between strain 13092 and two other strains, 14001 and 14003, isolated in the same location at one-year interval.

A pneumonic lung sample from a CCPP suspicion in the west of Niger was analysed at LABOCEL in 2019 for Mccp isolation and identification. DNA extracted from the seeded culture, amplified by universal 16S rDNA PCR, yielded an unambiguous Sanger-sequence, an indication that the sample was not containing multiple mycoplasma species. BLASTn analysis resulted in the identification of Mccp, with 99.7% identity to Mccp genomes, while only 97.9% to *M. capricolum* subsp. *capricolum* and 87.9% to *M. feriruminatoris* genomes. The DNA sample (ref: 19146) was then sent for Illumina sequencing. The mean GC% of the sequenced reads was 45%, which did not fit the known GC% of an Mccp genome (23.7%). When the reads were assembled on a reference Mccp genome, most of them (98%) where not assembled. Consequently, it was not possible to reconstruct a whole Mccp genome, although the reads that assembled confirmed that Mccp DNA was present in the sample. The unassembled sequences were used to perform a *de novo* assembly (data not shown) and the obtained contigs were analysed by BLASTn. They showed homology with goat DNA, an indication that the sample from which DNA was extracted was composed mainly of host cells.

Due to the small number of Mccp reads available for Niger-19146, this strain was analysed in a second step together with all available data. This resulted in the determination of a smaller number of informative SNPs (N = 396) which was nevertheless sufficient to obtain a phylogenetic tree and a position for that strain (Figure 1b). It shared the same node as 95043 (87.8 SHT value), the other Niger strain isolated in 1995. However, it was positioned at the extremity of a longer branch from that node, due to additional SNPs.

Representative Mccp whole genomes were obtained for each of the clusters previously defined by Dupuy et al., (2015), notably those of strains 04012 (CP040917) for group A, 05021 (CP041700) for group B; 91106 (CP041703) for group C and 07033 (CP041712) for group E. Reference genomes were already available for the other two groups: M1601 (CP017126) for group D and Abomsa (LM995445) for group F. Nine additional complete Mccp genomes were then obtained by performing assemblies on these “cluster-representative” genomes (Table 1). It was not possible to obtain complete genomes for all the assemblies, as some gaps remained that could not be resolved by our purely *in silico* approach.

## 4 Discussion-conclusion

A straightforward whole genome sequence analysis pipeline and phylogenetic analysis was adopted. This new pipeline naturally lead to a much higher number of SNPs being detected, 2880, compared with 239 with the previous strategy (Dupuy et al., 2015), and therefore much more robust phylogenetic trees and exquisite strain typability. It confirmed the phylogenetic groups that had been determined previously (Dupuy et al., 2015) on a limited number of genes. It allowed also the distinction of three samples of the same strain, Gabes, which had undergone various numbers of *in vitro* passages. This new strategy gave us some insight into the molecular epidemiology of CCPP in four geographical locations, Niger, Tanzania, United Arab Emirates and Asia, leading to epidemiological hypothesis that merit further studies.

The presence of CCPP in East Africa had long been suspected (Mettam, 1929). It was confirmed in Kenya by the *princeps* publication by MacOwan (MacOwan and Minette, 1976) and later in Ethiopia (Thiaucourt et al., 1992a) Uganda (Bolske et al., 1995) and in Tanzania in 1998 (Msami et al., 1998). One peculiarity for East Africa was that two lineages of Mccp strains were identified there (Pettersson et al., 1998) (Dupuy et al., 2015). The strict identity of the six Tanzanian 2013-Mccp genomes was therefore quite unexpected as they originated from various locations, some of them 400km apart (Figure 2b). This could indicate that a particularly virulent Mccp strain was circulating in East Africa around 2013. In fact, strain ILRI181, isolated the previous year in Kenya, which is the closest phylogenetic neighbour to the 2013-Tanzanian strains, has been shown to be highly virulent, leading to 100% morbidity in a challenge model (Liljander et al., 2019). The close proximity of some Mccp strains from Tanzania with those of Kenya or Uganda had already been noticed (Kusiluka et al., 2001) and can easily be explained by nomadism in that region that favours contacts and long range transmissions.

The fact that CCPP affects wildlife species has been proven since 2007 (Arif et al., 2007) but the number of wildlife Mccp isolates remained scarce up to now. Analysing four Mccp strains isolated from three different species was an interesting opportunity. All Mccp genome sequences obtained from wildlife in the Middle East clustered together in the phylogenetic tree (Figure 1a). At first, this could suggest some kind of adaptation to new hosts. This adaptation was observed with other mycoplasma species (Delaney et al., 2012). However, our sampling was biased, as we could not include Mccp strains isolated from goats in the region around the same time. New identification and sequencing efforts should be made to re-examine this hypothesis. Epidemiological data in the Middle East indicated that wildlife was rather being infected due to close contacts with goats (Lignereux et al., 2018). The fact that Mccp strains, isolated from wildlife, 31km apart (Figure 2c) clearly differ, also points toward that explanation.

The presence of CCPP in Asia was suspected a long time ago (Kwong, 1947) but it was confirmed only in 2007 (Li et al., 2007). Whole Mccp genome analysis revealed some interesting facts. Two Chinese Mccp strains, 87001 and zly1309, appeared very distantly related to the other Asian strains, including M1601, which is also of Chinese origin, and 12002 from Tajikistan. In the case of 87001 this could have been the result of numerous *in vitro* passages since its first isolation was claimed to be 1958 (Li et al., 2020). This cannot be the case for zly1309 which is a recent isolate. These results indicate that there could be a wide variety of Mccp strains circulating in Asia. Here again there is a need for a more representative number of Asian isolates for a comprehensive analysis of Mccp strains evolution.

The presence of CCPP in West Africa has never been clearly asserted. Local “inoculation” procedures indicate that pleuropneumonia in goats was present at the end of the XIXth century (Payton, 1887). But CCPP confirmation was only obtained with serological tools that were not proven strictly specific (Rurangirwa et al., 1990). Studying a CCPP suspicious sample from West-Niger was a unique opportunity. In fact, this sample was not included in the general Mccp phylogenetic tree due to the low number of available sequencing reads. A separate analysis with this sample showed that it positioned at the extremity of a branch, with strain 95043 as the closest relative. This is in agreement with the hypothesis that the 2019 strain results from the evolution of the Mccp strain isolated 24 years previously and 700km to the East, in Gouré in 1995 (Lorenzon et al., 2002). This may be an indication that CCPP is actually progressing westwards. Lefèvre had hypothesised this westward expansion of CCPP in Africa in 1987, when CCPP was confirmed for the first time in Chad (Lefevre et al., 1987). A molecular dating analysis had then determined that the most recent common ancestor to Central African Mccp strains had emerged around 1965 (Dupuy et al., 2015). The genome sequencing results of the 2019 Niger isolate are compatible with this hypothesis.

CCPP has long been a neglected disease. This may be explained by the fact that the most threatening goat disease is PPR, that a laboratory confirmation for CCPP is difficult and good quality CCPP vaccines not available or used in the field (Wambura et al., 2014). Nevertheless, reports from various countries indicate that antibiotic treatments are often administered to goats suffering from respiratory infections, whatever the cause. With the onset of a global eradication campaign against PPR (http://www.gf-tads.org/ppr/progress-on-ppr-control-and-eradication-strategy/en/) it is hoped that this burden will rapidly wane. This could lead to the emergence of other diseases, such as CCPP, or at least make the disease more visible. This situation is not unlike what happened in cattle in regards to contagious bovine pleuropneumonia once rinderpest was eradicated.

The isolation of Mccp in pure culture appears to be one of the biggest limitations to CCPP outbreak confirmation, when PCR cannot be performed locally. This limitation is not due to the paucity of Mccp in the samples but to its fastidiousness. On the contrary, pleural fluid samples contain very high densities of Mccp, more than 10^9^ color changing units/ml (Liljander et al., 2019), which should allow their purification with differential centrifugations and elimination of host inflammatory cells. In such a case, the extraction of Mccp DNA from pleural fluid would not only allow safer transport of biological samples but also its direct sequencing. This could be the method of choice for an international surveillance system which would not only permit the characterization of Mccp but also the detection of other pathogens, through a metagenomic approach.

Whole genome sequencing has become the gold standard for high-resolution typing method, which supersedes all previous phenotypic or genotypic methods, that could be applied for major public health pathogens (Seth-Smith and Egli, 2019; Mutreja and Dougan, 2020). This technique is particularly well suited for Mccp whose genome is not prone to recombination events. The WGS analysis pipeline described here will easily accommodate additional samples to the core genome alignment, and become a useful international portable surveillance procedure in the near future. Its primary use will be the analysis of transmission pathways and spatiotemporal analysis of CCPP. However, additional laboratory efforts to obtain complete whole genomes and metabolomics results for the whole set of strains, could provide valuable information when integrated into more comprehensive epidemiological databases. Information on strain virulence would be particularly helpful for genome data mining and deciphering the basis for virulence traits that are still elusive for Mccp.

## Declarations

### Author contribution statement

Etienne Loire, François Thiaucourt: Conceived and designed the experiments; Performed the experiments; Analyzed and interpreted the data; Wrote the paper.

Abdoulkarim Issa Ibrahim, Louis Lignereux: Contributed reagents, materials, analysis tools or data.

Lucía Manso-Silván: Analyzed and interpreted the data; Wrote the paper.

### Funding statement

This research did not receive any specific grant from funding agencies in the public, commercial, or not-for-profit sectors.

### Competing interest statement

The authors declare no conflict of interest.

### Additional information

No additional information is available for this paper.
